# Tofacitinib in the treatment of skin and musculoskeletal involvement in patients with systemic sclerosis, evaluated by ultrasound

**DOI:** 10.1007/s00296-021-04956-7

**Published:** 2021-07-27

**Authors:** Rositsa Valerieva Karalilova, Zguro Anastasov Batalov, Tanya Lyubomirova Sapundzhieva, Marco Matucci-Cerinic, Anastas Zgurov Batalov

**Affiliations:** 1 Department of Internal Diseases, Medical University of Plovdiv, Plovdiv, Bulgaria; 2 Rheumatology Clinic, University Hospital “Kaspela”, Plovdiv, Bulgaria; 3 Department of Experimental and Clinical Medicine, University of Florence, Rheumatology Section, Florence, Italy

**Keywords:** Tofacitinib, JAK/STAT, Systemic sclerosis, Ultrasound

## Abstract

Systemic sclerosis (SSc) is a rare autoimmune connective tissue disease characterized by fibrosis of the skin and internal organs, autoimmunity-driven damage and vasculopathy. The current approved disease-modifying treatments have limited efficacy, and treatment is guided toward alleviating organ complications. Thus, there is an unmet need for discovering new effective treatment options. There is recent evidence that the JAK/STAT signaling pathway is markedly activated in SSc patients. To assess the efficacy and safety of tofacitinib (TOF) on skin and musculoskeletal involvement as compared to methotrexate (MTX) in systemic sclerosis (SSc). In this 52-week pilot study, 66 patients with SSc were enrolled: 33 patients received 5 mg of oral TOF twice a day; 33 received 10 mg of MTX weekly. The proportion of dcSSc and lcSSc patients was similar (dcSSc: 42% TOF group and 36% MTX group; lcSSc: 58% TOF group and 64% MTX group). The primary outcome was the change in the modified Rodnan skin score (mRSS). Secondary outcomes included ultrasound (US) skin thickness and musculoskeletal involvement (US10SSc score). Digital ulcers (DUs) and adverse events (AEs) were documented through the treatment. Both groups had similar characteristics and medians on the outcome measures at baseline. At week 52, the TOF median mRSS was significantly lower than the MTX (*p* < 0.001) with a mean reduction of 13 points versus MTX 2.57. The mean percent improvement in the TOF group was 44% higher than in the MTX group. TOF median US skin thickness was significantly lower than MTX (*p* < 0.001), with a mean reduction of 0.31 mm versus 0.075 mm in the MTX group. The US10SSc median score was significantly lower in the TOF group (*p* = 0.002); mean reduction of 10.21 versus 5.27 in the MTX group. Healing of DUs with no new occurrences was observed in the TOF group. There was no significant difference between the groups in the number of AEs from baseline to week 52. TOF showed greater efficacy than MTX in reducing mRSS, skin thickness and musculoskeletal involvement in SSc and a satisfactory safety profile.

## Introduction

Systemic sclerosis (SSc) is a rare autoimmune connective tissue disease characterized by fibrosis of the skin and internal organs, autoimmunity-driven damage and vasculopathy [1]. SSc has the highest morbidity and mortality of all rheumatic diseases [2]. The current approved disease-modifying treatments have limited efficacy, and treatment is guided toward alleviating organ complications [3]. Thus, there is an unmet need for discovering new effective treatment options [4, 5].

There is recent evidence that the JAK/STAT signaling pathway is markedly activated in SSc patients [6]. In genetic studies, STAT locus variants have been shown to be strongly associated with SSc [7]. Therefore, JAK/STAT signaling may have crucial role in the pathogenesis of SSc.

Tofacitinib (TOF) inhibits JAK1/JAK3 and has been proven effective in rheumatoid arthritis (RA), psoriatic arthritis, ulcerative colitis, and a new therapeutic option in systemic lupus [8]. However, data regarding the use of JAK-inhibitors in patients with SSc are quite scarce. Blocking JAK/STAT activity with TOF abrogates core fibrotic responses in fibroblasts, and prevents multiple organ fibrosis in mice. These findings are the first to demonstrate that in SSc patients with genomic evidence of enhanced JAK/STAT pathway activity in target organs, TOF treatment might be effective in slowing or reversing fibrosis [9].

The aim of our pilot study was to assess the efficacy and safety of TOF treatment on skin and musculoskeletal involvement as compared to methotrexate (MTX) treatment in SSc patients.

## Methods

### Trial design and participants

This was an pilot study with two treatment arms, carried out between 2018 and 2020. The participants were 66 SSc patients of age ≥ 18 years at the time of consent. Among them 40 patients were with limited cutaneous SSc (lcSSc) and 26 with diffuse cutaneous SSc (dcSSc) [10] according to the 2013 classification criteria of SSc at least 24 weeks before screening [11].

The SSc patients were allocated to the TOF and MTX treatment groups by simple random assignment. The proportion of dcSSc and lcSSc patients was similar (dcSSc: 42% TOF group and 36% MTX group; lcSSc: 58% TOF group and 64% MTX group). Their characteristics at baseline were compared to rule out significant differences with confounding effect on the outcome of the treatments (Table 1).

**Table 1:** Baseline patient characteristics

Variables	Group		*p*
	TOF (*N* = 33)	MTX (*N* = 33)	
Age, mean (± SD) years	48.45 (± 12.35)	48.21 (± 11.51)	0.935^t^
Female, *n* (%)	30 (91%)	29 (88%)	1.000^f^
Diffuse cutaneous SSc, *n* (%)	14 (42%)	12 (36%)	0.801^f^
Limited cutaneous SSc, *n* (%)	19 (58%)	21 (64%)	0.801^f^
Disease duration, months!			
Mean (± SD)	34.69 (± 16.03)	34.54 (± 15.01)	0.969^t^
Interstitial lung disease (ILD), *n* (%)	13 (39.4%)	11 (33.3%)	1.000^f^
Pulmonary arterial hypertension (PAH), *n* (%)	5 (15.2%)	6 (18.2%)	0.745^f^
Gastrointestinal tract involvement (GIT), *n* (%)	19 (57.6%)	21 (63.6%)	0.801^f^
ANA positive, *n* (%)	30 (91%)	31 (93%)	1.000^f^
Anti–Scl-70 positive, *n* (%)	5 (15%)	4 (12%)	1.000^f^
Anti–RNA polymerase III positive, *n* (%)	3 (9%)	2 (6%)	1.000^f^
ACA positive, %	9 (27%)	10 (30%)	1.000^f^
CRP mg/l, mean ± SD	4.51 (2.22)	4.11 (1.64)	0.502^t^
Background immunosuppressive therapy, *n* (%)	0%	0%	na
Mycophenolate mofetil, *n* (%)	0%	0%	na
Azathioprine, *n* (%)	0%	0%	na
Use of prednisone, *n* (%)!!	1 (3%)	2 (6%)	1.000^f^
Escape therapy, *n* (%)	0 (0%)	2 (6%)	0.492^f^

*F*—Fisher’s exact test, *t*—*t* test for independent samples; !—from the time of first non-Raynaud’s phenomenon manifestation; !!—Prednison ≤ 10 mg; na—not applicable

Patients fulfilled the following inclusion criteria: 2013 ACR/EULAR classification criteria [11]; disease duration ≤ 5 years (from the first non-Raynaud phenomenon manifestation); modified Rodnan Skin Score (mRSS) ≥ 10 and less than 30 at screening visit; oral corticosteroids (CSs) ≤ 10 mg/day of prednisone or equivalent if the patient was on a stable dose regimen for ≥ 2 weeks prior to and including the baseline visit; ability to provide written informed consent.

Exclusion criteria were: rheumatic disease other than SSc; any serious bacterial infection within the last 3 months or any chronic bacterial infection; current treatment with hydroxychloroquine, D-Penicillamine, mycophenolate mofetil (MMF) or cyclophosphamide (CYC); current or prior history of treatment within the 3 months prior to baseline with biological disease-modifying anti-rheumatic drugs (bDMARDs); subjects at risk for tuberculosis (TB), according to the local guidelines for TB risk assessment; positive for HBV or for HCV at or within 30 days of screening; history of HIV; current or recent history of uncontrolled clinically significant disease; pregnant or breastfeeding female subjects; significant deviation of any of the laboratory results at screening; history of recurrent herpes zoster or disseminated herpes zoster or herpes simplex; history of any malignancy in the last 5 years with the exception of adequately treated basal cell or squamous cell skin cancer or cervical cancer in situ.

The study was approved by the Local Ethics Committee of the University Hospital “Kaspela” (Date: 22.05.2018; №58B). Written informed consent was obtained from all individual participants included in the study. The study was performed in accordance with the Good Clinical Practice Guidelines and with the World Medical Association Declaration of Helsinki, revised in 2000, Edinburgh.

### Endpoints

The *primary endpoints* were:

- Efficacy—the observed change in mRSS from baseline to week 52.
- Safety—the number of patients with at least one adverse event (AE), an AE leading to withdrawal or a serious AE (SAE). Safety assessments consisted of AEs documentation, physical examination, vital signs, laboratory evaluations and ECGs.

The *secondary endpoints* were the changes in Ultrasound (US) skin thickness and US joint and tendon score (US10SSc) [12]. DUs were documented as the exploratory efficacy endpoint.

### Dosing and visits

MTX dose was 10 mg/week. The TOF treatment included 5 mg twice daily. Rescue therapy with withdrawal of the study medication was permitted at principal investigator discretion.

#### Clinical evaluation

The clinical evaluation combined the following measures: the number of tender and swollen joints, the duration of morning stiffness, the Visual Analogue Scale (VAS) (0–100 mm) for patient’s and physician’s global assessment of disease activity (VAS PtGA; VAS PhGA). The presence of tendon friction rubs (TFRs), digital ulcers (DUs) and joint contractures was also documented at each visit. Throughout the study, mRSS skin involvement was measured by the same physician trained in the skin scoring technique [13]. The physician who performed the mRSS evaluation was blinded to the treatment received.

#### Ultrasound assessment

US assessment of the skin and joints and tendons was conducted by two assessors (RK—EULAR Ultrasound Trainer and TS—EULAR Advanced Level) who were blinded to the patients’ clinical data and type of treatment. US GE Logic E9 (18 MHz) was used for the skin assessment (by RK) and Esaote MyLab7 for the joint/tendon assessment (by TS) using multi-frequency linear probe (10–18 MHz).

### Musculoskeletal ultrasound (MSUS)

MSUS of both hands was performed using a multi-frequency linear probe (10–18 MHz). We examined 10 joints of both hands: wrist joint, second and third metacarpophalangeal (MCP) and second and third proximal interphalangeal (PIP) joints [12]. All ten joints and their adjacent tendons were sonographically assessed in a standardized manner and scored according to the OMERACT definitions of pathology [14]. All joints and tendons were assessed by GSUS and PDUS.

*GSUS*. The wrist joint was assessed for synovitis and tenosynovitis on dorsal, palmar and ulnar scan. Palmar scan was used to assess MCP2 and MCP3 for synovitis and tenosynovitis; and dorsal scan for paratenonitis. PIP2 and PIP3 were assessed for synovitis on palmar scan. Synovitis on GSUS was scored on a semi-quantitative scale (grade 0–3) [15]. Tenosynovitis/paratenonitis was documented as present (1) or absent (0).

*PDUS* was used for grading of synovitis and tenosynovitis/paratenonitis on dorsal and palmar scan for each joint. Synovitis and tenosynovitis/paratenonitis on PDUS were scored on a semi-quantitative scale (grade 0–3) [16, 17]. The scoring range was 0–30 for GSUS synovitis, 0–14 for GSUS tenosynovitis/paratenonitis, 0–66 for PDUS synovitis and 0–42 for PDUS tenosynovitis/paratenonitis. *US10SSc* score (range 0–152) was calculated as the sum of the synovitis score and tenosynovitis/paratenonitis score on GSUS; and of the synovitis and tenosynovitis scores on PDUS [12].

### Skin ultrasound

Skin thickness including the dermis was assessed by an experienced rheumatologist who was blinded to the clinical and MSUS data of the subjects [18–20]. The US system (GE E9) was equipped with an 18-MHz probe and all subjects were examined at five anatomical sites: the middle part of the proximal phalanx of the 2nd finger of the dominant hand (DH) dorsally; the area between the 2nd and 3rd MCP joint of the DH; the forearm area 4 cm proximal to the wrist joint of the DH; the lateral part of the lower leg 14 cm proximal to the ankle joint; 3 cm distal to the upper part of the manubrium sterni. The skin US assessment was performed in the longitudinal and transverse plane, in the same session and under same environmental conditions. Average values of regional skin thickness and total skin thickness (TST) were measured at two assessments and were recorded in millimeters, respectively. The skin thickness measurement on the predilection anatomical sites were marked before the assessment to minimize variations.

### Statistical analysis

Assuming 40% difference in mRSS change between the treatments, 95% CI, and 80% power, we estimated the minimum sample size at 27 patients per treatment. Descriptive statistics included means and standard deviations (± SD) for continuously measured and normally distributed variables, and medians and interquartile ranges (IQR) for non-normally distributed measurements. Between-treatment comparisons were established through the Mann–Whitney *U* test, and the 95% CI for the difference in medians (Hodges–Lehmann Median Difference). The change in the outcome variables was compared through an independent-samples t-test. Fisher’s exact test was used with categorical variables and for the comparison of proportions. All tests were two-tailed. The analyses were performed through the IBM SPSS V.27 software.

## Results

### Patient characteristics

Sixty-six patients were enrolled in the trial: 33 received oral TOF 5 mg twice daily; 33 received 10 mg weekly oral MTX. During the treatment period, three patients discontinued because of AEs. One patient in the TOF group withdrew due to progressive interstitial lung disease (ILD) (switched to CYC, followed by MMF), and two patients in the MTX group withdrew due to elevated transaminase levels (more than 2.5 times the upper limit of normal). The withdrawals were treated as missing data without replacement. Two patients in the MTX group and none in the TOF group received rescue therapy after week 26. The baseline characteristics of the patients were similar in the two treatment groups (Table 1).

### Cross sectional-treatment comparisons on the primary and secondary efficacy endpoints

Prior to the start of the treatment, both groups of patients had similar median scores on all three outcome measures, with no significant differences. At weeks 26 and 52, significantly lower medians were observed in the TOF group in comparison with the MTX group (Table 2).

**Table 2:** Outcome measures at baseline, the 26th and 52nd weeks

Outcome measures	Group	*N*	Median	IQR	Difference in medians (95% CI of difference)!	Mann–Whitney *U* *p*
Baseline						
mRSS	TOF	33	24.00	10.50	1.00 (− 3.00–4.00)	0.594
	MTX	33	23.00	10.00		
US skin thickness	TOF	33	1.71	0.52	0.02 (− 0.13–0.16)	0.822
	MTX	33	1.69	0.50		
US10SSc score	TOF	33	16.00	12.50	0.00 (− 4.00–4.00)	0.962
	MTX	33	16.00	10.50		
26th week						
mRSS	TOF	33	12.00	7.50	− 9.00 (− 11.00 to − 5.00)	< 0.001
	MTX	33	21.00	8.00		
US skin thickness	TOF	33	1.49	0.31	− 0.12 (− 0.27 to − 0.01)	0.040
	MTX	33	1.61	0.52		
US10SSc score	TOF	33	7.00	6.50	− 7.00 (− 8.00 to − 2.00)	< 0.001
	MTX	33	14.00	10.50		
52nd week						
mRSS	TOF	32	10.00	7.00	− 10.00 (− 12.00 to − 7.00)	< 0.001
	MTX	31	20.00	7.00		
US skin thickness	TOF	32	1.33	0.35	− 0.27 (− 0.37 to − 0.11)	< 0.001
	MTX	31	1.60	0.50		
US10SSc score	TOF	32	7.50	6.75	− 5.50 (− 7.00 to − 1.00)	0.002
	MTX	31	13.00	10.00		

*MTX* methotrexate, *TOF* tofacitinib, *mRSS* modified Rodnan skin score, *US* skin thickness—assessed by ultrasound, *US10SSc* ultrasound joint and tendon score, *IQR* interquartile range, ! Hodges–Lehmann Median Difference

### Change in mRSS

**Fig. 1:**
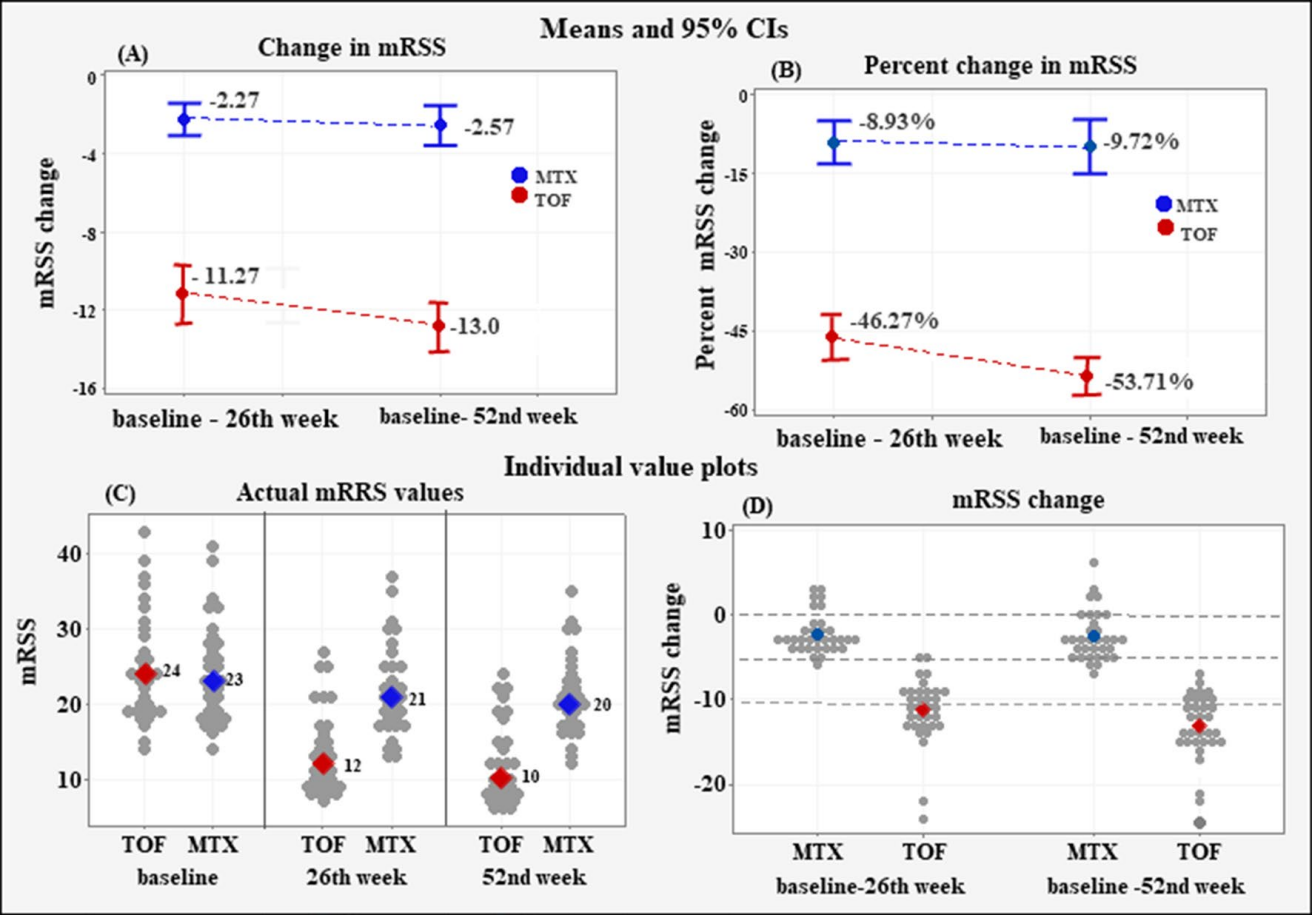
Mean change in mRSS (panel **A**); Mean percent change in mRSS (panel **B**); Individual value plots of the actual mRSS values (panel **C**); Individual value plots of the change in mRSS (panel **D**). The negative sign (−) denotes a reduction in the measurement from baseline to the 52nd week; 0 denotes no change; positive values denote an increase in the measurement

The mean change and the mean percent change in the mRSS score at the 26th week of treatment (Fig. 1) showed a higher reduction in the TOF treated patients (− 11.27 ± 3.89) as compared to the MTX treated patients (− 2.27 ± 2.32); difference − 9 (95% CI − 10.57 to − 7.42), *p* < 0.001 (**A**). The corresponding mean percent change (**B**) was − 46.27 ± 10.76% in the TOF group versus − 8.93 ± 11.53% in the MTX group; difference—37.34% (95% CI − 42.81% to − 31.84%), *p* < 0.001.

At the 52nd week, the TOF group mean change in mRSS was − 13.0 ± 3.48 versus − 2.57 ± 2.88 in the MTX group, difference − 10.42 (95% CI − 11.99% to − 8.85%), *p* < 0.001 (**A**). The mean percent change amounted to − 53.71 ± 10.36% in the TOF group and − 0.9.72 ± 14.63% in the MTX group; difference—43.99% (95% CI:  = 50.22% to − 37.75%), *p* < 0.001 (**B**).

The individual changes in mRSS at the 26th week ranged between − 5 and − 24 in the TOF group and between 3 and – 6 in the MTX group. At the 52nd week, the TOF individual reductions in mRRS varied between − 7 and − 24 as the majority of the patients (75%) improved beyond − 10 points. In comparison, the MTX individual changes varied between 6 and − 7, with 64% being below − 5 points, 13% without improvement, and 13% with an increase in mRSS (**C–D**).

### Change in US skin thickness

**Fig. 2:**
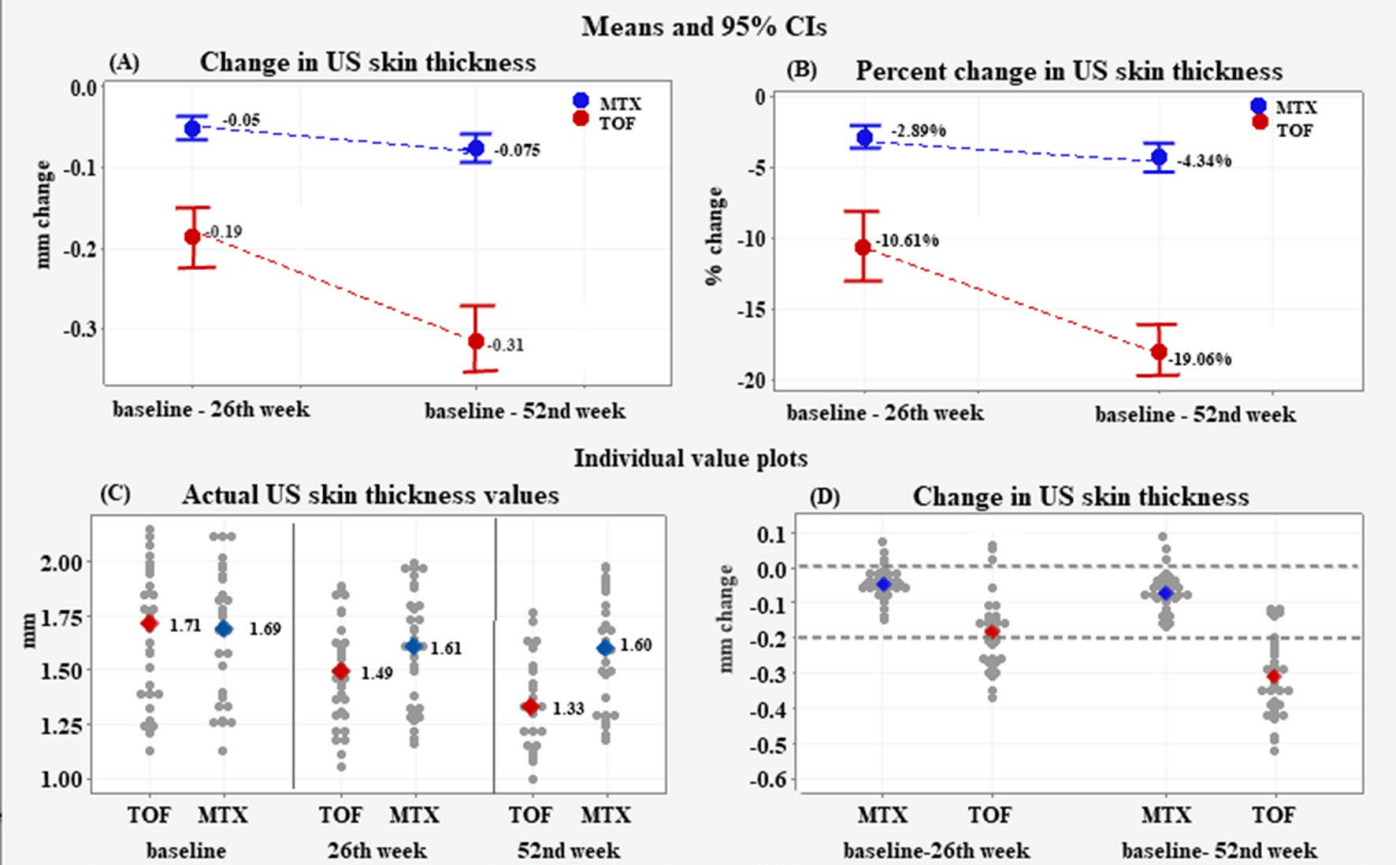
Mean change in US skin thickness (panel **A**); Mean percent change in US skin thickness (panel **B**); Individual value plots of the actual US skin thickness (panel **C**); Individual value plots of the change in US skin thickness (panel **D**). The negative sign (−) denotes a reduction in the measurement from baseline to the 52nd week; 0 denotes no change; positive values denote an increase in the measurement

At the 26th week, the mean reduction in US skin thickness (Fig. 2) for the TOF group was − 0.19 ± 0.02 mm versus − 0.05 ± 0.04 mm in the MTX group, difference − 0.13 (95% CI − 0.17 to − 0.090), *p* < 0.001 (**A**). The mean percent change in the TOF group amounted to − 10.61 ± 6.01% versus − 2.89 ± 2.22%; difference − 7.72% (95% CI − 9.93% to − 5.48%), *p* < 0.001 (**B**).

At the 52nd week, the TOF mean reduction in US skin thickness was − 0.31 ± 0.11 mm versus MTX − 0.075 ± 2.22 mm, difference—0.235 mm (95% CI − 0.27 to − 0.19), *p* < 0.001(**A**). The mean percent change at the 52nd week was − 19.06 ± 4.62% in the TOF group and − 4.34 ± 2.84% in the MTX patients; difference 14.72% (95% CI − 15.59 to − 11.82), *p* < 0.001 (**B**).

At the 26th week, the TOF individual changes in skin thickness ranged between 0.06 mm to − 0.37 mm vs. 0.1 mm to − 0.015 mm in the MTX group. At week 52, the TOF individual changes extended from − 0.12 mm to − 57 mm as the majority of the patients (88%) achieved reductions above − 0.20 mm. In comparison, the individual MTX changes remained within the range of − 0.05 mm to − 0.17 mm, with the majority of the reductions (85%) being below 0.10 mm (**C–D**).

### Change in US10SSc score

**Fig. 3:**
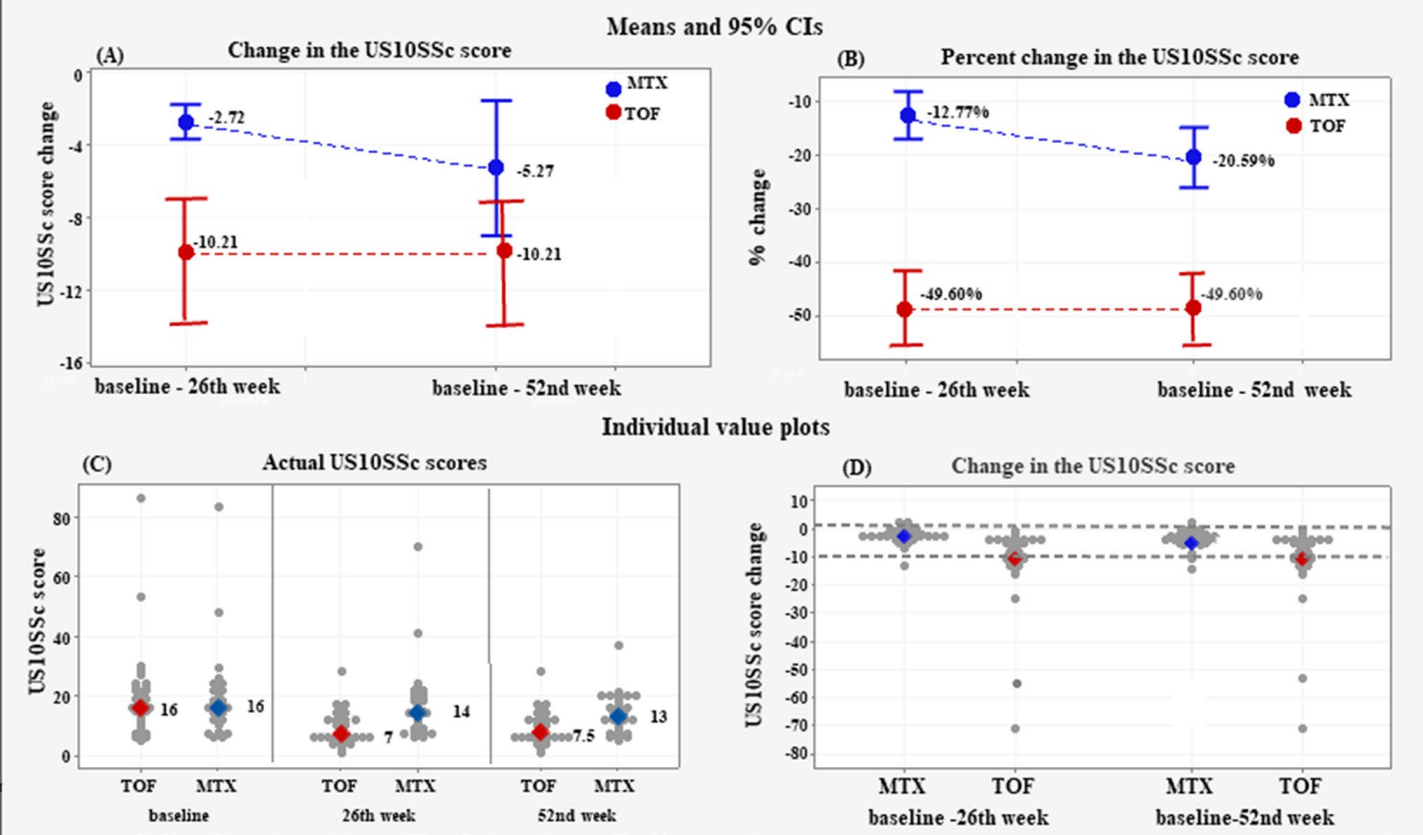
Mean change in the US10SSc score (**A**); Mean percent change in the US10SSc score (**B**); Individual value plots of the actual US10SSc scores (**C**); Individual value plots of the change in the US10SSc score (**D**). The negative sign (−) denotes a reduction in the measurement from baseline to the 52nd week; 0 denotes no change; positive values denote an increase in the measurement

At the 26th week, the TOF group achieved a mean decrease in the US10SSc score of − 10.21 ± 10.9 versus − 2.72 ± 2.72 in the MTX group (Fig. 3); difference 5.59 (95% CI − 7.61 to − 3.55), *p* = 0.001 (**A**). The mean percent decrease was 49.60 ± 17.30% in the TOF group versus 12.80 ± 12.50% in the MTX group; difference of 36.79% (95% CI − 42.75 to − 20.21), *p* < 0.001 (**B**).

Although the TOF patients did not experience further decrease in the US10SSc score at the 52nd week, whereas the MTX patients did, the effectiveness of the TOF treatment remained significantly better as shown by the difference of − 5.10 points (95% CI − 10.49 to − 0.61) in the mean US10SSc score change (− 10.21 ± 9.50 TOF versus − 5.27 ± 9.10 MTX, *p* = 0.030). The percent change was − 49.60 ± 17.30% in the TOF group versus − 20.59 ± 15.70% MTX; difference 29.01% (95% CI − 36.76 to − 20.59%), *p* < 0.001.

At the endpoint, the individual changes in the US10SSc score in the TOF group ranged between − 1 and − 1 points vs. 2 to − 13 points in the MTX group (**C–D**).

### Change in DUs

**Fig. 4:**
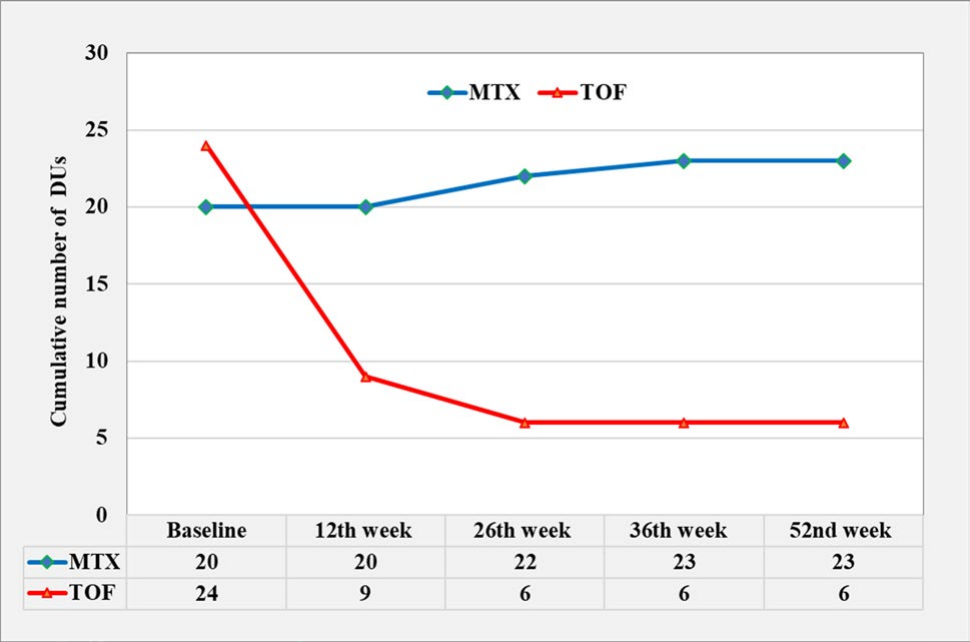
Change in the cumulative number of DUs

At baseline, DUs were documented in eight TOF and six MTX patients (Fig. 4). In the course of the treatment no new DUs developed in the TOF patients, and the total count of DUs was reduced by 75%. In the same group, a patient with long-term pruritus showed a substantial improvement. In comparison, no healing of DUs was observed in the MTX group and three new DUs occurred (15% increase).

### Safety endpoints

AEs were rare in both treatment groups (Table 3). A total of four SAEs were recorded of which one TOF patient who developed progressive ILD and withdrew from the study; two patients from the MTX group who withdrew due to elevated transaminase levels (more than 2.5 times the upper limit of normal), and one patient in the MTX group was diagnosed with basal-cell skin carcinoma at week 52.

**Table 3:** Adverse events (AEs) by week 52

Adverse events by the 52nd week	Group exact test		Fisher’s *p*
	TOF (*N* = 33)	MTX (*N* = 33)	
One or more AE, *n* (%)	11 (33%)	11 (33%)	na
One or more infectious AE, *n* (%)	4 (12%)	6 (18%)	0.733
One or more SAE, *n* (%)	1 (3%)	3 (9%)	0.613
SAEs leading to withdrawal, *n* (%)	1 (3%)	2 (6%)	na
Infections	4 (12%)	6 (18%)	0.733
Pneumonia	0 (0%)	0 (0%)	na
Viral infections	3 (9%)	5 (15%)	0.708
Herpes Zoster	0 (0%)	0 (0%)	na
Urinary tract infection	1 (3%)	0 (0%)	na
Infected DUs	0 (0%)	1 (3%)	na
Cardiac disorders	0 (0%)	2 (6%)	0.492
Atrial fibrillation	0 (0%)	1 (3%)	na
Acute coronary syndrome	0 (0%)	0 (0%)	na
Pericardial effusion	0 (0%)	1 (3%)	na
DVT	0 (0%)	0 (0%)	na
Lung disorders	1 (3%)	0 (0%)	na
Progressive ILD (SAE)	1 (3%)	0 (0%)	na
Pulmonary embolism	0 (0%)	0 (0%)	na
Gastrointestinal disorders	6 (18%)	2 (6%)	0.258
Cholecystitis	0 (0%)	0 (0%)	na
Increased level of cholesterol (> 1.5x)	2 (6%)	0 (0%)	0.492
Elevated levels of transaminases (> 1.5x- < 2.5x)	4 (12%)	0 (0%)	0.114
Elevated levels of transaminases (> 1.5x- < 2.5x)	0 (0%)	2 (6%)	0.672
Malignant disorders	0 (0%)	1 (3%)	na
Basal-cell skin carcinoma (SAE)	0 (0%)	1 (3%)	na

*na* not applicable

## Discussion

Skin involvement in diffuse SSc is a predictor for the extent of visceral involvement, prognosis and mortality [21]. MRSS reflects disease progression and its improvement is a positive prognostic sign and vice versa [22]. For these reasons, mRSS was selected as the primary efficacy endpoint. In addition, we evaluated musculoskeletal involvement because articular and periarticular involvement have been proven to be associated with more aggressive disease course, progression of skin fibrosis, internal organ involvement and worse prognosis [23, 24]. Furthermore, synovitis and TFRs are independent predictors for progression of skin fibrosis and ILD [25, 26].

From our results, we can extrapolate that TOF is more effective for reducing skin and musculoskeletal involvement than MTX. Both at the 26th and 52nd week, the TOF treated patients had significantly lower median values, and significantly higher mean reductions and mean percent reductions on the outcome variables as compared to the MTX group (Table 4).

**Table 4:** Summary statistics about the higher efficacy endpoint outcomes of the TOF treatment

Endpoint outcome	26th week		52nd week	
	TOF	MTX	TOF	MTX
mRSS				
Mean change	− 11.27	− 2.27	− 13.0	− 2.57
Mean % change	− 46.27%	− 8.93	− 53.71%	− 9.72%
Range of change	− 5 to − 24	3 to − 6	− 7 to − 24	6 to − 7
US skin thickness (mm)				
Mean change	− 0.19	− 0.05	− 0.31	− 0.075
Mean % change	− 10.61%	− 2.83%	− 19.06%	− 4.34%
Range of change	0.06 to − 0.37	0.1 to − .015	− 0.12 to − 0.57	− 0.05 to − 0.17
US10SSc score				
Mean change	− 10.21	− 2.73	− 10.21	− 5.27
Mean % change	− 49.60%	− 17.77	− 49.60%	− 20.59%
Range of change	− 1 to − 71	2 to − 13	− 1 to − 71	2 to − 13

In the planning phase of the study, we assumed a 40% better outcome on the primary measure (mRSS) in the TOF treatment group as compared to the MTX group. At the 26th week, a difference of 9 points in mean mRSS reduction and a difference of 37.34% in mean percent reduction were observed in favor of TOF. At the 52nd week, these differences increased further to 10.43 points in mean mRSS reduction and 44% in mean percent reduction. The TOF individual changes ranged from − 7 to − 24 points, with the majority (75%) achieving a reduction of over − 10 points. In comparison, the individual changes in the MTX group varied between 6 and − 7 points as 64% were below − 5 points, 13% did not show change, and 13% showed an increase in mRSS.

The results of other recently published randomised controlled trials with tocilizumab [27, 28], abatacept, riociguat and belimumab in diffuse SSc were close to statistical significance, but did not meet the primary endpoint of reduction in mRSS [27–32]. In the faSSccinate trial, at week 48 the difference in the mRSS between the two groups was − 3.55; in the focuSSced trial − 1.73 [27, 28]. In the RISE trial the mean difference between the riociguat and placebo groups at week 52 was − 2.34 [29, 32], whereas at the end of the open-label extension the mean improvement in mRSS was − 3.02 in the riociguat–riociguat group and − 3.96 in the placebo–riociguat group [33]. In the ASSET trial for abatacept, the mean change in the mRSS at month 12 was − 4.49 points for the placebo group and − 6.24 points for the abatacept group, not reaching statistical significance [30]. In the belimumab trial, a statistically significant improvement was observed with a median change in mRSS of − 10 in comparison to the placebo group − 3.0, but clinical significance was not reached [31].

The assessment of skin thickness by mRSS has some disadvantages concerning its objectivity, bias among assessors and inability to detect subtle skin change. Other techniques like high-frequency US are more sensitive, objective and reliable for the evaluation of skin thickness [34–36]. Increasing skin thickness measured by US indicates increasing disease severity [37]. The extent of the mean reduction in skin thickness in the TOF group in our study attests to its higher level of effectiveness as compared to MTX. The mean reduction was by 0.235 mm and by 14.72% higher in the TOF group. The individual reductions in the TOF group varied within a larger range (− 0.12 mm to 0.57 mm) versus − 0.02 mm to − 0.17 mm in the MTX group.

Musculoskeletal US may be used to assess articular and periarticular involvement in SSc patients [38–41]. Elhai et al. showed that signs of tendon involvement were more frequent in SSc than in RA patients [42]. Sclerosing tenosynovitis, detected by US as hyperechoic tendon sheath thickening, appeared to be more specific for SSc and infrequent in RA [42–44].

In our study, TOF was more effective than MTX for reducing musculoskeletal involvement in SSc. In fact, the mean decrease of joint and tendon US score (US10SSc score) was significantly higher in the TOF group at both time points.

The TOF treatment showed a positive effect on the number and size of DUs and was overall well tolerated. No difference was observed in the number of AEs in both treatment groups, and no cases of herpes zoster or deep venous thromboses were detected in the TOF treated group. Discontinuation rates (3% for the TOF group and 6% for the MTX group) were lower in the present study as compared to the trial with tocilizumab (9%) [28].

Our pilot study is one of the first trial about the efficacy of TOF treatment for patients with SSc. The employment of US to objectively measure and monitor the efficacy of TOF and MTX over skin and musculoskeletal involvement is another novel aspect. The fact that all three outcome measures (mRSS, US skin thickness and US10SSc score) showed consistent results may corroborate the validity of the observed clinical ameliorations.

However, there are also some limitations. First, this was an open-label study, which has an inherent weakness of biasing the results towards the expected outcome. We must also recognize the relatively small sample size, which can be explained by the small population of SSc patients in our country, estimated as approximately five cases in 10 000 individuals. Finally, the duration of exposure to TOF was relatively short (52 weeks), which may have limited the safety assessment to the period under observation.

## Conclusion

TOF demonstrated greater efficacy than MTX in reducing mRSS, skin thickness and musculoskeletal involvement in SSc patients. TOF has also shown a satisfactory safety profile.

## Data Availability

All data relevant to the study are included in the article.

## Declarations

### Patient and public involvement

Patients and/or the public were not involved in the design, or conduct, or reporting, or dissemination plans of this research.
